# Evidence for the amnion-fetal gut-microbial axis in late gestation beef calves^1^

**DOI:** 10.1093/tas/txaa138

**Published:** 2020-12-22

**Authors:** Gwendolynn L Hummel, Kelly L Woodruff, Kathleen J Austin, Travis L Smith, Hannah C Cunningham-Hollinger

**Affiliations:** 1 Department of Animal Science, University of Wyoming, Laramie, WY; 2 Laramie Research and Extension Center, University of Wyoming, Laramie, WY

## INTRODUCTION

The existence of the placental microbiome in humans has been championed (Aagaard et al., 2014) and opposed (de Goffau et al., 2019) in recent years, leading to ambiguous conclusions regarding its effects, if any, on fetal gut development in utero. Although this ambiguity continues in ruminants, the microbiome of the fetal membranes may be integral to the development of the calf rumen microbiome, where it could potentially affect long-term performance.

Current research indicates that microbial inoculation occurs prior to birth in humans (Stinson et al., 2019) and ruminants (Alipour et al., 2018), suggesting the fetal membranes as a likely microbial niche supplying the gut of the growing fetus. We hypothesize that the calf is exposed to a microbial source prior to birth and that the amnion, as the most intimate fetal membrane in relation to the fetus, plays a role in facilitating this inoculation. Well-established relationships exist between the rumen microbiome and feed efficiency (Shabat et al., 2016; Li and Guan, 2017), and the research suggests that there is programming potential within the rumen microbiome (Abecia et al., 2014; Yáñez-Ruiz et al., 2015). Understanding these early inoculation mechanisms may lead to long-term impacts on the production efficiency in the host.

We hypothesize that the calf rumen is exposed to a microbial source prior to birth and that the amnion plays an essential role in its inoculation. Our objective was to isolate and identify bacterial and archaeal species in the late gestation amniotic fluid (AF), and compare these species to the rumen fluid (RF) of the newborn calf prior to suckling, as well as the meconium (MEC) of the neonate. Vaginal swabs (VAG) were collected alongside AF, in order to be evaluated as a contributing source of rumen microbe inoculation.

## MATERIALS AND METHODS

Experimental protocols were approved by the University of Wyoming Institutional Animal Care and Use Committee.

### Sample Collection

Multiparous Angus cross-bred cows (*n* = 10) were bred via artificial insemination, and VAG samples were collected 10 d prior to the expected calving date utilizing sterile, double-sheathed equine uterine culture swabs (Jorgenson Labs, Loveland, CO). As described by Bicalho et al. (2017), VAG samples were collected prior to amniocentesis following a caudal epidural and disinfection of the vulva and perineal area. Each swab was exposed from the sheath at a midpoint of the vaginal cavity, rotated for maximum contact at least six times, and returned to the sheath. All AF samples were collected via amniocentesis under caudal epidural anesthesia (Garcia and Salaheddine, 1997).

Immediately following parturition, RF and MEC were collected from each calf before being allowed to suckle. Methods for oral lavage collection of RF are described by Zhou et al. (2009). Briefly, a stomach tube is lubricated and passed orally to the rumen, where suction is applied via syringe, and 20 to 30 mL of ruminal fluid is aspirated. A sterile, double-sheathed equine uterine culture swab was utilized to collect MEC samples immediately following parturition (Alipour et al., 2018). Each swab was inserted into the rectum of the newborn calf, the cotton tip exposed, and the swab rotated at least six times before retraction into the sterile sheath and removal from the rectum. All samples were immediately placed on dry ice and stored at −80 °C.

### Microbial DNA Isolation and Sequencing

Fluid samples were processed as 0.25-g aliquots in bead tubes containing sterilized zirconia (0.3 g of 0.1-mm beads) and silicon (0.1 g of 0.5-mm beads), along with 1 mL of lysis buffer (500 mM NaCl, 400 mM Tris–HCl, 50 mM ethylenediaminetetraacetic acid, 4% sodium dodecyl sulfate; Yu and Morrison, 2004). Swab samples were processed under the same protocol by sterilely cutting and placing whole swab heads in their respective bead tubes. Due to insufficient sample volume, fewer AF samples were processed (*n* = 8). Chemical and mechanical lysis was performed on all samples prior to DNA extraction utilizing the Precellys Evolution (Bertin Instruments, Rockville, MD; Zhao et al., 2015). Isolation included a 70 °C incubation, repetition of lysis steps utilizing 300 µL of lysis buffer, and centrifugation to precipitate (Yu and Morrison, 2004). Microbial DNA was further purified utilizing the QIAmp DNA Stool Minikit (Qiagen, Santa Clara, CA) for amplicon 16S rRNA sequencing. DNA purity and concentration was determined using Nanodrop. Aliquots of 30 ng/µL of each sample were processed for sequencing following the Illumina MiSeq System library protocol, where the hypervariable region V4 of the 16S rRNA gene was sequenced.

### Bioinformatics Analysis

Utilizing QIIME2 v. 2019.10 (Bolyen et al., 2019), quality filtering, denoising, and pairing were accomplished via the DADA2 plugin (Callahan et al., 2016). Taxonomic classification was performed using the pretrained 16S 515F/806R classifier from the Silva 132 database (Yilmaz et al., 2014) and visualized in QIIME2 based on relative abundance. Shannon index, evenness, and Faith’s phylodiversity indices for alpha diversity were generated in QIIME2 and sample type compared using Kruskal–Wallis permutational multivariate analysis of variance (PERMANOVA). Beta diversity sample-type pairwise comparisons were also determined using PERMANOVA, and diversity was analyzed using the Bray–Curtis dissimilarity matrix and visualized using principle coordinate analysis. Statistical significance was considered when *q* ≤ 0.05, and a tendency toward significance was considered when 0.05 > *q* ≤ 0.10, with *q* representing each *P*-value adjusted for false discovery rate.

## RESULTS

### Alpha Diversity

**Figure 1.**
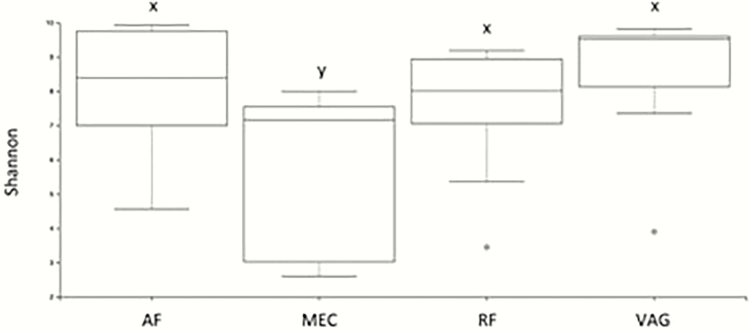
Alpha diversity box plots showing Shannon richness for amniotic fluid (AF), meconium (MEC), rumen fluid (RF), and vaginal swabs (VAG). x,y denote tendencies to differ at 0.05 > *q* < 0.10.

Abundance and evenness of microbial taxa are accounted for in the Shannon diversity index (Fig. 1), where AF and MEC displayed a tendency toward dissimilarity (*q* = 0.09), as well as MEC and RF (*q* = 0.09) and MEC and VAG (*q* = 0.06). No other sample types displayed a tendency toward dissimilarity with another (*q* ≥ 0.14). Evaluation of evenness revealed a similar trend, with no two sample types displaying significant differences from another (*q* ≥ 0.37).

**Figure 2.**
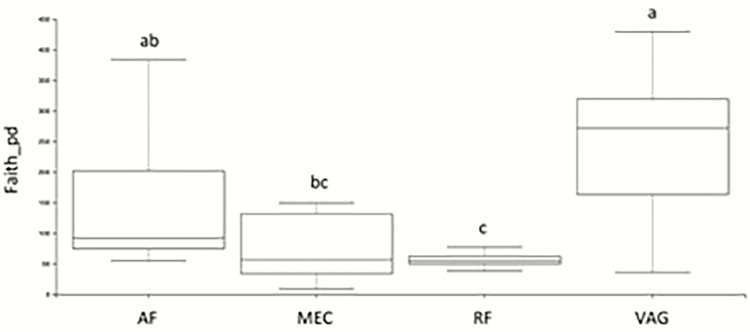
Alpha diversity box plots showing Faith’s phylogenetic diversity for amniotic fluid (AF), meconium (MEC), rumen fluid (RF), and vaginal swabs (VAG). a,b,c denote significant differences at *q* ≤ 0.05.

Faith’s phylogenetic diversity (Fig. 2) measures biodiversity regarding phylogenetic differences between microbial species. Pairwise comparisons revealed MEC and VAG to differ significantly (*q* = 0.03), but not MEC and AF (*q* = 0.40). In addition, RF differed from both the AF and VAG (*q* = 0.03), but not MEC (*q* = 1.00). No differences were reported between AF and VAG (*q* = 0.43).

### Beta Diversity

Under the Bray–Curtis dissimilarity matrix, which is utilized to determine compositional dissimilarities between samples, only MEC and RF were not significantly different (*q* = 0.92). However, MEC and VAG were different (*q* = 0.003), as were RF and VAG (*q* = 0.007). The AF differed from MEC, RF, and VAG (*q* = 0.003).

## DISCUSSION

As an essential proxy for the in utero gut microbiome in humans (Walker et al., 2017), MEC samples are utilized to represent microbial colonization events in late gestation. Although MEC tended to differ from VAG and AF under Shannon index, Faith’s phylogenetic diversity indicated a significant difference in phyla between MEC and VAG, but not MEC and AF. Importantly, the observed similarity between MEC and AF alpha diversity may indicate a crucial relationship between the AF and colonization of the fetal gut. This may be due to the fact that a gestating fetus consumes AF during regular development (Walker et al., 2017), thereby introducing AF microbial populations to the fetal gut prior to parturition. This mechanism may facilitate the amnion-fetal gut-microbial axis as a pivotal method of in utero microbiome inoculation.

Because the MEC and VAG samples differed in Faith’s phylogenetic and beta-diversity measurements, and tended to differ in Shannon index, it is likely that microbial ascension from the vagina plays a lesser role in fetal gut inoculation in late gestation. However, AF and VAG were found to differ only in beta diversity. In cattle, uterine microbial taxa have been observed to become less diverse during gestation (Moore et al., 2017), and in humans, vaginal microbes have been identified in uterine tissue (Witkin, 2014). The lack of dissimilarities in alpha-diversity measurements could also indicate a contribution of the vaginal microbiome to AF, while allowing AF to play the major role in fetal gut colonization, as a more intimate tissue to the fetus.

## IMPLICATIONS

These results indicate that the calf rumen is indeed exposed to a microbial source prior to birth and confirm findings regarding microbial inoculation in the human gut. Previous investigations indicate the AF as a likely source of microbes for the developing gut. These results provide further evidence for the existence of the amnion-fetal gut-microbial axis. As such, these data indicate a critical role of the amniotic microbiome in late gestation, while also introducing a role for vaginal microbes in the maturation of the calf’s microbiome postpartum. Further understanding is required of the maternal influence on the development of the reproductive and calf rumen microbiome, allowing maternal nutrition and reproductive management in beef cows to be conducted in a way that programs the optimal rumen microbiome for calf performance.
